# Rothmund-Thomson syndrome

**DOI:** 10.1186/1750-1172-5-2

**Published:** 2010-01-29

**Authors:** Lidia Larizza, Gaia Roversi, Ludovica Volpi

**Affiliations:** 1 Department of Medicine, Surgery and Dentistry, University of Milan, Italy; 2 National Cancer Institute, Milan, Italy; 3 Department of Biology for Medical Sciences, University of Milan, Italy

## Abstract

Rothmund-Thomson syndrome (RTS) is a genodermatosis presenting with a characteristic facial rash (poikiloderma) associated with short stature, sparse scalp hair, sparse or absent eyelashes and/or eyebrows, juvenile cataracts, skeletal abnormalities, radial ray defects, premature aging and a predisposition to cancer. The prevalence is unknown but around 300 cases have been reported in the literature so far. The diagnostic hallmark is facial erythema, which spreads to the extremities but spares the trunk, and which manifests itself within the first year and then develops into poikiloderma. Two clinical subforms of RTS have been defined: RTSI characterised by poikiloderma, ectodermal dysplasia and juvenile cataracts, and RTSII characterised by poikiloderma, congenital bone defects and an increased risk of osteosarcoma in childhood and skin cancer later in life. The skeletal abnormalities may be overt (frontal bossing, saddle nose and congenital radial ray defects), and/or subtle (visible only by radiographic analysis). Gastrointestinal, respiratory and haematological signs have been reported in a few patients. RTS is transmitted in an autosomal recessive manner and is genetically heterogeneous: RTSII is caused by homozygous or compound heterozygous mutations in the *RECQL4 *helicase gene (detected in 60-65% of RTS patients), whereas the aetiology in RTSI remains unknown. Diagnosis is based on clinical findings (primarily on the age of onset, spreading and appearance of the poikiloderma) and molecular analysis for *RECQL4 *mutations. Missense mutations are rare, while frameshift, nonsense mutations and splice-site mutations prevail. A fully informative test requires transcript analysis not to overlook intronic deletions causing missplicing. The diagnosis of RTS should be considered in all patients with osteosarcoma, particularly if associated with skin changes. The differential diagnosis should include other causes of childhood poikiloderma (including dyskeratosis congenita, Kindler syndrome and Poikiloderma with Neutropaenia), other rare genodermatoses with prominent telangiectasias (including Bloom syndrome, Werner syndrome and Ataxia-telangiectasia) and the allelic disorders, RAPADILINO syndrome and Baller-Gerold syndrome, which also share some clinical features. A few mutations recur in all three RECQL4 diseases. Genetic counselling should be provided for RTS patients and their families, together with a recommendation for cancer surveillance for all patients with RTSII. Patients should be managed by a multidisciplinary team and offered long term follow-up. Treatment includes the use of pulsed dye laser photocoagulation to improve the telangiectatic component of the rash, surgical removal of the cataracts and standard treatment for individuals who develop cancer. Although some clinical signs suggest precocious aging, life expectancy is not impaired in RTS patients if they do not develop cancer. Outcomes in patients with osteosarcoma are similar in RTS and non-RTS patients, with a five-year survival rate of 60-70%. The sensitivity of RTS cells to genotoxic agents exploiting cells with a known *RECQL4 *status is being elucidated and is aimed at optimizing the chemotherapeutic regimen for osteosarcoma.

## Disease names and synonyms

Rothmund-Thomson syndrome (RTS) (OMIM#268400)

Poikiloderma atrophicans and cataract

## Definition

RTS is an autosomal recessive genodermatosis presenting in infancy with a characteristic facial rash (poikiloderma), the diagnostic hallmark, and heterogeneous clinical features including short stature, sparse scalp hair, sparse or absent eyelashes and/or eyebrows, juvenile cataracts, skeletal abnormalities, radial ray defects, premature aging and a predisposition to osteosarcoma, a malignant tumour originating in bone.

RTS was originally described in 1868 by the German ophthalmologist Rothmund who observed poikiloderma, growth retardation and rapidly progressive bilateral juvenile cataracts in 10 children in a Bavarian village [1]. In 1936, the English dermatologist Thomson reported three similar patients with "Poikiloderma congenitale" and growth retardation who displayed skeletal defects, including bilateral thumb aplasia and hypoplastic radii and ulnae, but no cataracts [2]. The eponym Rothmund-Thomson syndrome was coined by Taylor in 1957 to describe a group of patients with the above-mentioned disorders [3]. At present the rationale for such grouping awaits further knowledge on the molecular basis of the syndrome.

Following the association in 1999 of a subset of RTS cases with homozygous or compound heterozygous mutations in the human helicase gene *RECQL4 *[4], two forms of RTS have emerged based on clinical and molecular analysis: type I RTS, characterised by poikiloderma, ectodermal dysplasia and juvenile cataracts, negative for the *RECQL4*-mutation scan and type II RTS, with poikiloderma, congenital bone defects and an increased risk of osteosarcoma related to deleterious *RECQL4 *mutations [5]. Whether RTS I and II represent distinct syndromes with overlapping clinical signs or intersecting nosological entities involving genes acting on the same pathway, remains to be assessed.

## Epidemiology

RTS is a very rare disease and reliable data on its prevalence are not available. To date, approximately 300 patients have been recorded in the medical literature [6-8]. Due to the highly variable clinical spectrum [9], which brings together patients with shared and unique developmental defects, patients who display an atypical/borderline clinical presentation may be overlooked. Consistent with autosomal recessive transmission, most patients appear as isolated cases but a few siblings, mostly from consanguineous families or from small close communities, have been reported [1,2,4,10-16]. The carrier frequency is unknown.

Whether RTS has a predilection for one sex over the other is unclear. An equal female-to-male ratio, a female predominance (1.4:1), and a male predominance (2:1) have all been reported in various case series [17]

RTS has been described in all ethnic groups: no founder effect has been detected in a specific population, although certain mutations may exist within defined populations.

## Clinical description

Patients may display few or many of the associated clinical features specified below, depending on their prevalence in the reported series of cases and the developmental origin of the affected tissue. The severity of each sign can also vary. The clinical suspicion of RTS is usually raised by the dermatologist because the ectodermal compartment is affected in most RTS patients. The first signs are those that affect the skin, hair, nails and teeth, which belong to the epidermis and the dermis and are of ectodermal and mesodermal origin respectively. Teeth, like hair and nails are epithelial appendages and their malformations are classified as cutaneous rather than as extracutaneous manifestations [18].

Growth delay and the resulting short stature is the second major clinical sign of RTS. The skeletal system is of extraordinary relevance to the pathogenesis of the disease, as shown by congenital skeletal defects in a consistent subset of patients and by more subtle anomalies, visualised on X-rays in the majority of the cases. Ocular lesions are currently considered a minor sign, possibly specific to a subset of RTS patients: although initially the prevalence of cataracts was reported to be as high as 50% in some series [6], it was subsequently found to be much lower [19]. Gastrointestinal, haematological and respiratory anomalies/clinical signs have been recorded sporadically. Cancer predisposition is a central sign in the development of the disease. It is not surprising that the most frequent tumour types, osteosarcoma (generally in childhood, or adolescence) and spinocellular carcinoma of the skin (in adults), develop in the cellular compartments most severely affected by the disease.

### Skin

**Figure 1:**
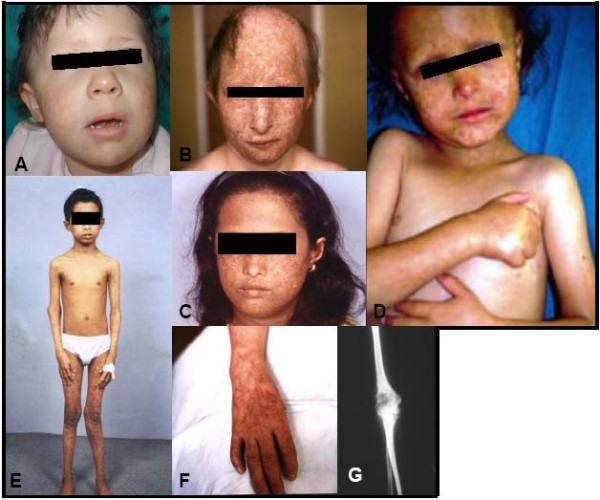
**Panel showing some clinical features of the RTS syndrome**. **A**) Chronic phase of cheek poikiloderma (4-year-old girl). **B**) Poikiloderma with alopecia (21-year-old boy). **C**) Poikiloderma. **D**) Poikiloderma sparing the trunk (courtesy of Professor M. Paradisi, Rome). **E**) Photo distributed poikiloderma and valgism of the knees. **F**) Thumb aplasia (patient B). **G**) Bone defect seen by X-Rays: *cystic*-like destructive lesion of the humerus (distal epiphysis) without apparent solution of continuity of the cortical bone (patient E).

The main clinical diagnostic hallmark of the syndrome is the cutaneous rash, which usually develops between the age of three and six months as erythema, swelling and blistering on the face (Fig. 1A), which subsequently spreads to the extremities (first on the extensor, then on the flexor surfaces) and to the buttocks. The trunk and abdomen are usually spared (Fig. 1D, E). The rash develops over time from the acute to the chronic phase with telangiectasic lesions, reticulated areas of depigmentation, hyperpigmentation and punctate atrophy. These changes, described as poikiloderma (Fig. 1B, C, D) [20] persist throughout life. Café au lait spots may develop later. Worsening of the erythema following sun exposure is experienced by some patients. Although 89% of the cases present within the first two years of life, late onset of poikilodermatous changes has been reported to occur. However, no mutation assay has been performed in these cases to suggest that they may be variant forms of RTS [21]. Poikiloderma occurs in a number of genodermatoses and other syndromes but with a subtly different pattern (see Differential Diagnosis). The accurate recognition of the onset, development and stabilisation of poikiloderma is thus necessary to establish the clinical suspicion of RTS.

A few subjects have early ageing of the skin. Palmo-plantar hyperkeratotic lesions occur in about one-third of individuals. The overall histological picture is diffuse lamellar hyperkeratosis.

Calcinosis cutis and porokeratosis are uncommon but have been reported [22].

### Hair

Sparse, brittle, thin or absent scalp hair and sparse hair on eyelashes and/or eyebrows were observed in 50% and 73% of the patients, respectively (Fig. 1A, B, D), in a cohort of 41 RTS patients evaluated in detail by Wang [19]. Beard, pubic and axillary hair may be almost absent and partial or total alopecia may develop (Fig. 1B).

### Nails

Dystrophic or poorly formed nails are common. Pachyonichia is also a frequent sign.

### Teeth

Dental abnormalities associated with RTS were first noticed by Rothmund in his original article [1]. They include a wide variety of malformations such as microdontia, rudimentary or hypoplastic teeth, multiple and unusual crown formations, and disorders of dental breakthrough. Structural defects of connective tissue have been shown by electron microscopy on a gingival biopsy specimen from one case [23]. Dental abnormalities affecting the complete permanent dentition, which were detected by radiographic examination, have been described in detail in an adult male patient with RTS. He had abnormally short roots, mainly of the maxillary teeth, and his third molars had only partially erupted [18]. An increased incidence of caries has been observed in RTS patients [24].

The overall incidence of dental abnormalities has been estimated at between 27% and 59% of the cases [6,9,18].

### Growth

Low birth weight, slow weight gain and linear growth deficiency are present in at least two-thirds of RTS patients [19]. RTS patients are proportionately small (for weight and height) without asymmetry. Short stature is defined as -2SD (standard deviation) score. Most patients with the characteristic short stature have normal growth hormone levels, but isolated cases with growth hormone deficiency have been described [9,25,26].

### Neurocognitive development

Neurologic cognitive milestones and intelligence are usually normal. Mental retardation was reported in six out of 202 patients [6]. Delayed speech development was found in a few cases [27]; Nathanson et al. [28] reported a case of poikiloderma congenitale with short stature and mental retardation. More recently Gelaw and coauthors [29] suggested that the accelerated cerebral atrophy, which was demonstrated in one case with RTS, Klippel-Feil syndrome and osteosarcoma, may be the underlying cause of mental retardation seen in a subset of patients with RTS.

### Deafness

Sensorineural deafness has been reported in one patient [30].

### Skeleton

In a review of the world literature [5,6], 68% of the patients were found to display skeletal anomalies, including frontal bossing, saddle nose and abnormalities of the long bones. The latter often appear as congenital radial ray defects, ranging from absence of one or both radii to short dysmorphic ulnae, absent or hypoplastic thumbs (Fig. 1F), hypoplasia/agenesis of the patella, syndactyly and diffuse or localised osteoporosis. In the clinical review of Wang, 75% of the patients, who underwent radiographic skeletal surveys, were found to have skeletal abnormalities that could not have been detected by clinical examination alone [19]. This percentage has been recently confirmed by a radiographic survey of 28 RTS patients who had undergone the *RECQL4 *mutation test [31]. The most common anomalies were abnormal metaphyseal trabeculation, brachymesophalangy, thumb aplasia or hypoplasia, osteopaenia, destructive bone lesions (Fig. 1G), dislocation of the radial head, radial aplasia or hypoplasia, and patellar ossification defects. Genotype-phenotype analysis showed that all patients with the *RECQL4 *mutation had skeletal abnormalities, further confirming that the *RECQL4 *mutational status may be indicative of increased risk of osteosarcoma.

### Ocular lesions

Bilateral cataracts, which are rapid in onset (usually 2 to 3 months) and are subcapsular, develop in the early years of life and represent the most frequent ocular sign, though they are not as common as previously indicated. Some affected patients go completely blind later in life. Other ocular abnormalities include exophthalmos, corneal atrophy/scleralization, congenital bilateral glaucoma [28], retinal atrophy/coloboma, strabismus, photophobia and blue sclerae. Iris dysgenesis has been reported in two instances [11,22,32].

### Gastrointestinal system

Oesophageal or pyloric stenosis, anal atresia, annular pancreas and rectovaginal fistula have been described [33]. Feeding problems may be encountered in infancy, with some patients requiring tube feeding. Gastrointestinal disturbances that manifest as chronic emesis and diarrhoea usually resolve in early childhood [26].

### Respiratory system

Lower respiratory tract infections have been rarely reported in patients with RTS [34]. Bronchiectasis was described in an RTS patient who had died of acute myeloblastic leukaemia [14]. Localised bronchiectasis with recurrent pneumonia that had developed without neutropaenia has been recently described in two cases of RTS [35]. Characterisation of *RECQL4 *mutations in one of the two patients supports the view that bronchiectasis is likely to be a novel feature of this rare syndrome [35].

### Haematological signs

Progressive leukopaenia and chronic microcytic hypochromic anaemia requiring transfusion have been reported [14,27]. Malignant haematological abnormalities ranging from myelodysplasia to aplastic anaemia and leukaemia have been identified [14,36-39].

### Cancer

A detailed review of the published work reporting on RTS patients who developed at least one neoplasm outlines the spectrum and age of onset of cancers to which the patients are predisposed [40]. Osteosarcoma occurs in childhood (mean age of onset: 14 years) whereas cutaneous epithelial neoplasms (squamous cell carcinoma, basal cell carcinoma and Bowen's disease) occur later (mean age of onset: 34.4 years). Both types of cancer develop at a younger age than would be normally expected. The estimated prevalences of osteosarcoma and skin cancer in RTS are 30% and 5%, respectively [19].

**Figure 2:**
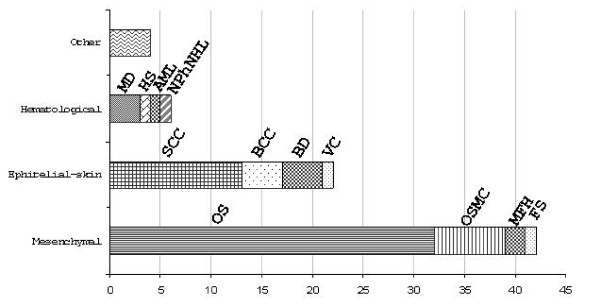
**Absolute number of tumours developed by RTS patients (X axis) grouped by the tissue of origin for each tumour type (Y axis): Among mesenchymal tumours, osteosarcoma (OS) is the most frequent**. Several cases of multicentric osteosarcoma (OSMC) have been described, while malignant fibrous histiocytoma (MFH) has been reported in two cases. Squamous cell carcinoma (SCC) is the most frequent cutaneous epithelial tumour followed by basal cell carcinoma (BCC) and Bowen's disease (BD). Myelodysplasia has been reported in three cases (MD). Reports of single cases: fibrosarcoma* (FS), verrucous carcinoma (VC), Hodgkin's "sarcoma" (HS), acute myeloid leukaemia (AML), nasopharyngeal non-Hodgkin's lymphoma* (NPhNHL). The group *other *consists of single cases of gastric carcinoma, malignant eccrine poroma *, parathyroid adenoma *, amelanotic melanoma (41) (*all these were reported as a second neoplasia).

The clustering of the two main cancer types can be seen in the histograms in Fig. 2, which shows the absolute number of tumours described in RTS patients (cases of secondary tumours, where the histological subtype is the same as for the first, have been omitted). Among mesenchymal tumours other than isolated osteosarcoma (OS), which represents the most frequent tumour, malignant fibrous histiocytoma (MFH) has been reported in more than one case. Squamous cell carcinoma (SCC) is the most common epithelial tumour. As far as haematological tumours are concerned, myelodysplasia has been reported in three cases [36,38,39]. Tumours that have been described only once are also included in Fig. 2[40,41].

Isolated osteosarcoma (OS) in RTS patients shows clinical features similar to those in patients with sporadic OS; for example the site of tumour development (most often the femur and the tibia) and the histological subtype (mainly osteoblastic). However, as in most cancer predisposing syndromes, the age of onset of OS in RTS patients is earlier than in the sporadic counterpart (14 *vs*. 17 years).

The abnormality in metaphyseal trabeculation in RTS patients, often observed on X-rays, has been considered intriguing as most osteosarcomas tend to originate in the metaphyses [31].

Besides the isolated osteosarcoma (OS), seven cases of multicentric OS (OSMC) have been reported [19,42-44]. Osteosarcoma is usually defined as multicentric if the tumour occurs at two or more sites in a patient in the absence of pulmonary metastases. A multicentric osteosarcoma is synchronous if more than one lesion is found at presentation and metachronous if development of apparently new tumour(s) is detected at a new site [45]. Considering this, three additional cases in which second primary osteosarcoma lesions have been described, apparently without pulmonary metastasis, may also be included as OSMC [9,46-48], making a total of ten cases. It has been reported that the incidence of multicentric disease associated with sporadic osteosarcoma ranges between 0.4% and 10% of all cases of osteosarcoma [49]. In comparison to this incidence in isolated OS in RTS, the proportion of OSMC is higher: 17.9 to 25.6%, based on cases that were not definitely assessed (overall 32 cases) [16,40]. This suggests an increased risk of OSMC in this syndrome. Similar considerations have been obtained from the evaluation of the incidence of metachronous OS in a group of twelve patients with OS [50].

Indeed, a notable proportion of RTS tumour carriers developed more than one tumour of the same or different type, showing that this is the main characteristic of a cancer predisposition syndrome. This is particularly true for a few patients who showed a very high frequency of recurrence of the disease. Significant examples are a patient with an adnexal skin tumour and malignant eccrine poroma, who developed eleven basal cell carcinomas [51] and another patient who developed seven skin tumours [40].

There is no evidence for increased cancer risk in the obligate heterozygous parents of RTS cases.

## Aetiopathogenesis

Type II RTS, characterised by poikiloderma and skeletal defects is caused by homozygous or compound heterozygous mutations in the *RECQL4 *(also named *RECQ4*) helicase gene [4]. Type I RTS, characterised by poikiloderma and juvenile cataracts is negative for the *RECQL4 *mutation and is one (or a set) of orphan Mendelian disease(s), for which the responsible gene(s) is (are) intensively sought but has (have) not yet been found. The *RECQL4 *gene, located at chromosome 8q24.3 [52], spans 21 exons [53] and its expression is regulated by a housekeeping promoter containing specific binding sites for several important transcription factors: SP1, AP1, AP2CRE and PAE3. It encodes a 133-kDa protein of 1208 aminoacids, RECQ protein-Like 4 (RECQL4; OMIM #603780), *i.e*. the ATP-dependent DNA helicase Q4, which contains the conserved DNA helicase domain homologous to the *Escherichia coli *RecQ helicase [54]. There are at least five distinct RECQ helicases in man that function at the interface of DNA replication, recombination and repair [55,56]. The RECQ helicases have received considerable attention during recent years due to their link to premature aging and cancer susceptibility syndromes. Mutations in three of these helicase genes, *RECQ2 (BLM), RECQ3 (WRN) *and *RECQL4*, result in the rare autosomal recessive disorders of Bloom, Werner and RTS, which share the general characteristics of genomic instability and cancer predisposition. However, each syndrome possesses unique clinical, cellular and genetic features that highlight the non-overlapping roles of the respective genes in the maintenance of genome integrity.

RECQL4 plays a role in a DNA-dependent ATPase activity and in single-stranded DNA annealing activity, but its helicase activity was doubtful [57,58] and as such RECQL4 appeared to be distinct from all other members of the RECQ helicase family. It has been demonstrated only recently that RECQL4 is not the only helicase-dead member of the RecQ family as two distinct regions of the protein, the conserved helicase motif and the N-terminal domain, each independently promote ATP-dependent DNA unwinding [59]. Localisation of the protein has been shown to be both nuclear and cytoplasmic [57]: nuclear localisation and retention domains are amino-terminal, unlike other RecQ proteins which show carboxyl-terminal nuclear localisation signals [60]. A cumulative set of data suggest that RECQL4 has roles in several different important cellular pathways [61]. In HeLa cells, the RECQL4 protein forms a stable complex with the ubiquitin ligases, UBR1 and UBR2, which are involved in the N-end rule pathway shown to be essential for correct chromosome segregation and apoptosis [57,62]. The RECQL4 protein is significantly expressed in the S-phase of the cell cycle, suggesting a role in DNA replication [63]. Indeed, RECQL4 has been shown in a *Xenopus *model to be important for initiation of DNA replication with its N-terminus being required for the recruitment of DNA polymerase **α **and its loading onto chromatin [64,65]. The implication of RECQL4 in replication initiation and cell growth has been demonstrated by mouse models showing that disruption of exons 5-8 (corresponding to the N-terminus upstream of the conserved helicase domain) results in embryonic lethality [66], whereas mice with deletion of either the single exon 13 or exons 9-13 (corresponding to helicase domain) are viable [67,68]. The possible involvement of RECQL4 in the repair of DNA double-strand breaks (DSB) has been demonstrated by the coincidence of RECQL4 nuclear foci with the foci formed by RAD51, a crucial protein which functions in homologous recombination of DNA DSB [69], as well as by the sensitivity of fibroblasts from RTS patients to ionizing radiation [6] and by the participation of RECQL4 in DSB repair in *Xenopus *egg extracts [70]. RECQL4 interacts with poly(ADP-ribose) polymerase-1 (PARP-1), which is involved in a competing end-joining pathway of DSB repair [71], in transcriptional regulation and in base excision repair (BER) [72]. This suggests that RECQL4 may act in these pathways or in a combination of these pathways. It has been reported that RECQL4 plays a role in oxidative stress and its amount increases in the nucleolus after treatment of cells with agents that induce reactive oxygen species (ROS) [73]. A defect in responding to ROS might explain the development of premature aging in RTS, as one of the hallmarks of aging is the accumulation of reactive oxygen species. RECQL4-deficient fibroblasts are hypersensitive to hydrogen peroxide-induced oxidative stress and show decreased cell proliferation and a reduction in DNA synthesis [74]. A role of RECQL4 in the repair of ultraviolet (UV)-induced DNA damage in human cells, through interaction with nucleotide excision repair factor Xeroderma Pigmentosum Group A (XPA), has been recently demonstrated [75].

Given the multiple roles of RECQL4 in DNA metabolism, it is likely in RTS patients with *RECQL4 *mutations that defective DNA replication, enhanced oxidant sensitivity and unrepaired DNA lesions would lead to sustained genomic instability. It has been noted that the function of RECQL4 may be especially important in a few proliferating tissues, such as developing bone and skin, as defects in RTS patients mainly affect these tissues [74]. Genotype-phenotype analysis shows that RTS patients with *RECQL4 *mutations are at a significantly higher risk of developing osteosarcoma as compared to RTS patients without *RECQL4 *mutations [5,8]. Indeed, at the cellular level, defects in RECQL4 manifest themselves as both numerical (mosaic trisomies) and structural (high frequency of rearrangements, particularly isochromosomes) chromosomal instability (CIN) and it is conceivable that accumulation of CIN can drive progenitor cells of sensitive cell lineages towards neoplastic transformation.

Mutations in the *RECQL4 *gene have been associated with two additional recessive disorders: RAPADILINO (Radial hypoplasia, Patella hypoplasia and cleft or Arched palate, DIarrhoea and dislocated joints, Little size and limb malformation, slender Nose and nOrmal intelligence), mainly observed in Finland [76] and Baller-Gerold syndrome (BGS), which is characterised by radial hypoplasia and craniosynostosis [77-79]. Although the three syndromes share some clinical features (*e.g*. short stature and radial ray abnormalities) there are also syndrome-specific features [80]. For example, cataracts are seen only in RTS, joint dislocation and patellar hypoplasia are seen only in RAPADILINO and craniosynostosis only in BGS.

**Figure 3:**
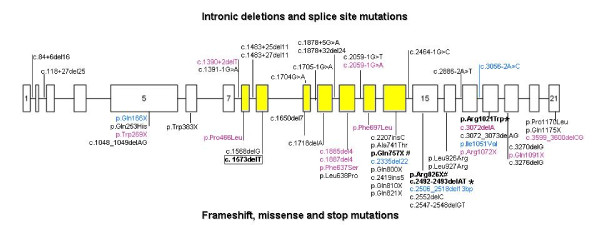
**Map of known mutations in the *RECQL4 *gene (exons are indicated by boxes and introns by interconnecting lines) in RTS (black), RAPADILINO (purple) and BGS (blue) patients**. Yellow identifies the exons encoding the helicase domain. Intronic deletions and splice site mutations are grouped above, while frameshift, missense and stop mutations are grouped below the graphic representation of the gene. Bold characters indicate mutations shared by RTS and RAPADILINO (#), RTS and BGS (*) and by RTS, RAPADILINO and BGS (framed).

To date, 56 different mutations in the *RECQL4 *gene have been identified, 39 of which are in RTS patients (see the comprehensive list provided by Siitonen [81] to which only a few mutations need to be added) [16,35,82]. The mutations found in all the three RECQL4 diseases are summarised in Fig. 3. The types of observed mutations are: i) nonsense mutations that change an aminoacid to a stop codon and lead to termination of protein translation; ii) insertions and/or deletions, which lead to reading frameshift and subsequent termination of protein translation; iii) mis-splicing alterations including substitutions at canonical splice junctions or at splice site consensus sequences that cause the skipping of exons and a downstream frameshift and subtle intronic deletions, which reduce intron size below the threshold (<80 bp) required for correct splicing [83-85] and iv) missense mutations that cause an aminoacid change in the protein. Most of the mutations identified in RTS are nonsense or frameshift mutations, which destabilise the mature mRNA through nonsense mediated-decay. Splice-site or missense mutations are usually present in combination with the former mutations. Splicing mutations are quite common: it has been suggested that both the G-C rich minisatellite flanking the 3' splice site of IVS12 in the helicase domain of the *RECQL4 *gene and exonic SNPs (Single Nucleotide Polymorphisms) belonging to high-score motifs for SR proteins may play a role in RECQL4 mRMA mis-splicing [86]. Splicing mutations also include deep intronic deletions, cryptic mutations identifiable only by RNA analysis, the harmful effect of which depends on the genomic structure of the *RECQL4 *gene. Indeed, 13 of the 21 introns of the *RECQL4 *gene are <100 bp and fortuitous multibase deletions make them unspliced, implicating intron-size constraint as a mutational mechanism in RTS [83]. These mis-splicing mutations are generally found in a homozygous state.

**Figure 4:**
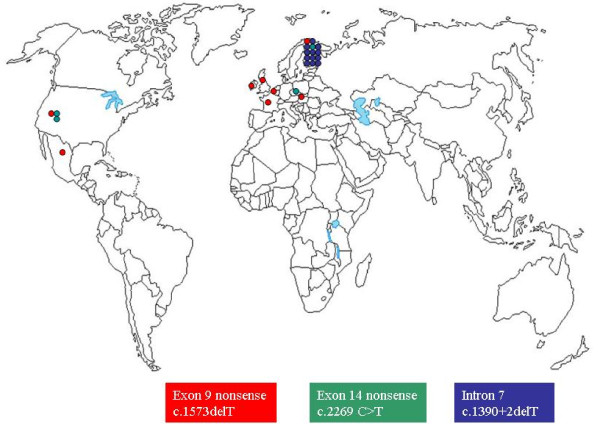
**Geographic distribution of recurrent mutations**.

There are a few recurrent mutations; the geographical distribution or clustering of the most significant ones is shown in Fig. 4. It is not surprising that the most common exon 9 c.1573delT (p.Cys525AlafsX33) *RECQL4 *mutation is shared by patients with the three distinct syndromes, RTS [12], RAPADILINO (one) and BGS (one). Compound heterozygotes with this and a second mutation from a large panel have been described among patients from several ethnic backgrounds [5,9,13,30,76,79,87-90](Additional file 1) indicating spreading of the haplotype on which c.1573delT had occurred. An associated SNP c.1568G>C, signalled in three RTS patients [30,89,90] and possibly overlooked by other studies, may be a part of the founder haplotype, probably Caucasian according to the ethnicity of the described RTS patients. No homozygous patient has been ever detected, raising the suspicion that this truncating mutation may have severe consequences. Indeed, we know by transcript analysis that the c.1573del T allele is not transcribed [30].

The second recurrent mutation shown on the world map (Fig. 4) is exon 14 nonsense c.2269C>T (p.Gln757X), which is shared by RTS and RAPADILINO. As in the above cases, this mutation is also widely distributed and, with a single exception, is found together with a large number of different mutations in patients of different ethnicity [5,9,16,76,87,88] (Additional file 1). Two additional mutations, exon 15 c.2476C>T (p.Arg826X) and intron 12 splicing c.2059-1G>T, the latter possibly enhanced by the contiguous unstable minisatellite [86], are found in both RTS and RAPADILINO.

The third recurrent mutation is the Finnish-specific intron 7 c.1390+2delT, mostly present in a homozygous state [76,91] (Additional file 1). So far, this splicing mutation has been detected only in RAPADILINO patients. The mutation is one of the Fin-major mutations (with a frequency in the Finnish population of 1/137 [76], with a prevalent founder mutation as the main contributor due to the population history of this very isolated country. With the exception of this, the promiscuity of *RECQL4 *mutations is remarkable. However, the combination of two different mutations appears to be critical for the determination of one of the three RECQL4 syndromes. Indeed, RTS and RAPADILINO or BGS patients have been found to share only one of the two mutations. This observation suggests that mutant *RECQL4 *alleles are primary determinants of the phenotype. However, there is the single exception of one RTS and one BGS patient who share both the earlier mentioned exon 9 nonsense c.1573del T p.Cys525AlafsX33 and the exon 18 missense p.Arg1021Trp mutations [79,90]. Due to the wide clinical expressivity of the RECQL4 syndromes, patients with the same genotype at the *RECQL4 *locus should be clinically re-evaluated to confirm their different clinical diagnoses. Such patients, and novel similar ones, might be a valuable resource to search for SNP-array genotyping variants acting as modulators of the phenotype.

## Genotype-phenotype correlations

A general view of the mutations underlying RTS predicts the expression of low levels of a truncated protein that lacks either the entire or a part of the helicase region. The complete loss of RECQL4 function is lethal in humans and all known mutant proteins found in patients are partially active [56]. This is due to the presence of the region near the N-terminus of the protein homologous to the proteins *Sld2 *in *S. cerevisiae *and *DRC1 *in *S. pombe*, which are necessary components for the initiation of DNA replication [64]. Localisation of nuclear localisation and retention activities to the RECQL4 amino-terminus would provide nuclear transport of putative truncated proteins encoded by RTS mutant alleles [60]. It thus remains possible that all RTS cell lines have residual RECQL4 activity. In keeping with this view, only two mutations have been detected upstream of exon 5: intron 1 c.84+6del16 and intron 2 c.118+27del25, both lead to mis-splicing and both are present in compound heterozygous patients (Fig. 3). Moreover, a deletion of the entire gene has never been identified. Mouse models also indicate that the N-terminal domain of RECLQ4 is crucial for cell growth. Mice with targeted disruption of *recql4 *exons 5-8 (corresponding to the N-terminus region upstream of the helicase domain) die between day 3.5 and 6.5 [66]. By contrast, mice with either a hypomorphic mutation, which deletes the single exon 13 [67] or a more extensive region of exons 9-13 [68], are viable and recapitulate the clinical symptoms of human RECQL4 diseases. In both cases, the mutant alleles express aberrant transcripts that could potentially be translated into defective Recql4 polypeptides, a situation similar to many mutant alleles identified in RTS II. The knockout mice of Hoki had a 5% survival rate by the age of two weeks and the survivors displayed severe growth retardation, bone defects and impeded cell division [67]. The low bone mass phenotype in these *Recql4-/- *and in the surrogate heterozygous *Recql4 +/- *mice appears to be associated with defects in osteoblast progenitors [92]. Parallel findings on control adult mouse bone that show Recql4 protein localisation in active osteoblasts around the growth plate but not in fully differentiated osteocytes, indicate that Recql4 has a function in the regulation of osteoprogenitor proliferation. Impairment of this cellular mechanism could underlie the skeletal anomalies common to all three defective RECQL4 syndromes [92]. Mice with deletion of the Recql4 helicase domain also show congenital skeletal defects, a distinctive skin abnormality and increased cancer susceptibility in a sensitised genetic background [68]. Interestingly, fibroblasts from the viable *Recql4 *mutant mouse lacking a functional helicase domain were found to display chromosomal instability, aneuploidy, a high frequency of spontaneous micronuclei and premature chromosome separation. This shows that a cohesion defect contributes to chromosomal instability [68]. The overall results from the three knockout models reveal that these mice present with a spectrum of signs that mimic the situation in humans, where different mutations in the *RECQL4 *gene lead to different phenotypes. Recent biochemical clues show that each of the two distinct regions of the protein, the conserved helicase motif and the *Sld2*-like-N-terminal domain, independently promotes ATP-dependent unwinding [59]. This, together with genetic studies in human RTS patients and in mouse models, demonstrates that RECQL4 is a multifunctional protein and each specific function may require different domains. Data on transcript analysis and on RECQL4 mutant proteins obtained by using antibodies specific for the N- and C-terminus would permit validation of the view that disruption of different regions of the RECQL4 protein affects different aspects of normal development and genome stability. Genotype-phenotype correlations by which the combination of two *RECQL4 *gene mutations can predict the phenome of the three RECQL4 syndromes should be addressed. Life expectancy is not impaired in RTS patients if they do not develop cancer. Thus, the most important genotype-phenotype correlation in RTS patients relates to the increased risk of cancer, especially osteosarcoma, which was thought to be determined by the presence of at least one truncating mutation [5]. Consistent with this assumption, the most common mutation in RAPADILINO syndrome, an exon 7 in-frame deletion that spares the helicase region, was found in patients without malignancies [76]. However, prolonged follow-up of RAPADILINO patients has revealed cancer in a few homozygous individuals, as well as in heterozygous carriers of this founder mutation [81]. Interestingly, green fluorescent protein-tagged constructs deleted for exon 7 show a cytoplasmic rather than a nuclear location, thus mapping the RECQL4 import domain to this region [60]. Recently a patient with BGS carrying two *RECQL4 *mutations in exon 15 was reported to develop a midline NK/T-cell lymphoma [82]. In keeping with these findings, an increased risk of cancer may be predicted for patients with RECQL4 mutations belonging to all RECQL4 syndromes. All *RECQL4*-negative RTS patients are not at an increased risk of cancer.

## Diagnosis

Due to the relatively non-specific nature of the symptoms, clinical diagnostic criteria and a score to establish a "definite", "probable" or "possible" diagnosis of RTS are not available. RTS is a disorder with onset in childhood and clinical diagnosis is currently based on the time of onset, spreading and the appearance of poikiloderma. According to Wang and Plon [8], diagnosis of probable RTS can be made if the rash is atypical and if two of the following clinical signs are present: sparse hair on the scalp, eyebrows and eyelashes, short stature and congenital bone defects (including subtle anomalies that are visible only on X-rays), dental and nail abnormalities, hyperkeratosis, cataracts, and cancers.

The diagnosis of RTS should be considered in all patients with osteogenic sarcoma, particularly if associated with skin changes [9]. The currently available molecular diagnosis, possibly combining different methods for analysing the *RECQL4 *gene should be made available to patients with typical poikiloderma and additional signs. Patients with a clinical presentation at the interface of two distinct RECQL4 syndromes [26,82,90] should also be tested for *RECQL4*.

The *RECQL4 *test is also recommended for RTS cases that share clinical signs with syndromes usually considered in the differential diagnosis either because of dermatological abnormalities or because they belong to the group of DNA repair disorders with chromosomal instability (see below).

Only two thirds of patients with a clinical diagnosis of RTS carry *RECQL4 *mutations. So far, no other gene has been detected that can account for the RTS patients without *RECQL4 *mutations. *RECQL4*-negative patients currently include a clinically and genetically heterogeneous group with RTSI patients, who like the RTSII, have poikiloderma but often display juvenile cataracts, and other RTS patients with atypical, borderline or unique phenotypic presentations.

## Differential diagnosis

The diagnosis of RTS can be difficult. Several cases have been identified only after they had developed cancer, especially osteosarcoma [50]. The onset of osteosarcoma in a young patient should raise the suspicion of a cancer predisposition syndrome such as retinoblastoma, Li-Fraumeni, Werner (see below) and RTS syndrome. In the absence of cancer, especially when other clinical signs are missing, the differential diagnosis begins with a dermatological examination and, if a chronic phase is established, the following causes of childhood poikiloderma should be taken in account [8,92,93].

• **Acrogeria **(Gottron syndrome), a premature skin aging syndrome, characterised by poikiloderma and lipoatrophy which affects mainly the distal extremities and manifests itself at birth or shortly afterwards. The skin becomes dry, thin, transparent and wrinkled, is easily bruised and exhibits telangiectasia. Hair and eyes are normal. Small stature has been reported in several patients.

• **Hereditary sclerosing poikiloderma **(AD), the main signs of which are a generalised poikiloderma appearing in childhood, with accentuation in flexural areas and on extensor surfaces and sclerodermatous plaques on palms and soles.

• **Dyskeratosis congenita **(XR, AD or AR) characterised by the triad: abnormal skin pigmentation (89% of patients), nail dystrophy (88%) and leukoplakia (78%). Skin changes are characterised by reticulated hyperpigmentation or hypopigmentation on the face, the neck, the trunk, and the thighs. The onset of poikiloderma and other clinical manifestations occurs generally during childhood, though later than in RTS. Severe nail involvement is the initial feature, which is followed by poikiloderma. Defective dentition and mental retardation are more commonly noted than in RTS. There is no photosensitivity. About 80-90% of patients develop bone marrow abnormalities by the age of 30.

• **Kindler's **syndrome (bullous acrokeratotic poikiloderma) (AR), is a hereditary bullous poikiloderma syndrome, where poikiloderma arises generally at the age of 2-3 years. Chronic trauma-induced blistering and photosensitivity, which usually start in early infancy, develop further. Indeed, the patients typically show acral bullae, which are present at birth or develop during the first few days of life, with an onset often resembling epidermolysis bullosa. The trauma-induced blistering and photosensitivity of early infancy improve with age giving place to poikiloderma, more prominent on the face and neck, and involving sun-exposed and non-sun-exposed skin, while skin atrophy is diffuse. Additional features include stenosis of the oesophagus, anus and urethra, webbing of digits, ectropion and chronic inflammation of oral mucosa, dental abnormalities and anhidrosis. A few of these signs may also be found in RTS patients.

• **Xeroderma pigmentosum **(AR), an accelerated photo-aging syndrome, with clinical signs limited to UV-exposed skin/tissue. Acute sun sensitivity from early infancy, characterised by severe sunburn with blistering or persistent erythema after minimal sun exposure, affects 50% of the patients. Numerous freckle-like hyperpigmented macules appear on sun-exposed skin in all individuals, and typically, when present on the face of a child before age two years, represent the hallmark of the disease. These abnormalities evolve into poikilodermatous changes in late childhood and give a greater than 1000-fold increased risk of cutaneous skin cancer. Ophthalmologic abnormalities are as frequent as cutaneous findings and are usually limited to the UV-exposed portion of the eyes: conjunctiva, cornea, and lids (no congenital cataract). Other signs include neurologic abnormalities (30% of patients).

• **Clericuzio type Poikiloderma with Neutropaenia (PN) **(AR). Poikiloderma on the face and limbs (starting in infancy) and short stature are features in common with RTS. However, unlike in RTS, the poikiloderma spreads from a rash arising on the distal limbs to the rest of the body, without sparing the flexural areas and trunk. Other particular signs of Clericuzio Poikiloderma are neutropaenia causing recurrent, especially pulmonary, infections, and pronounced hyperkeratotic nails, especially of the toes. No photo or heat sensitivity has been reported [94,95]. The gene for Clericuzio type Poikiloderma with Neutropenia (PN) has been recently identified [96], confirming the distinct genetic control of PN and RTS.

• **Exocrine pancreatic hypofunction and atrophy of the pancreas **have been recently described in a 20-year-old male with many clinical features of RTS, but no mutation in the *RECQL4 *gene [97]. Due to marked blister formation, the initial differential diagnosis included epidermolysis bullosa (EB) and Kindler syndrome (KS). The authors considered this case to be a peculiar variant of RTS.

Other rare genodermatoses, with prominent telangiectasias but not true poikiloderma, also need to be differentiated from RTS. They comprise a panel of DNA repair defects, in particular Bloom's syndrome and Werner syndrome, which are caused by defects in two other RECQ helicases and are also characterised by chromosomal instability.

• **Bloom's syndrome **(AR) is characterised by skin lesions (patchy areas of hypo- or hyperpigmentation) that are caused by chronic exposure to the sun. These develop from a red sun-sensitive telangiectatic erythema, which appears during the first or second year of life, into a "butterfly distribution" on the face (similar to lupus erythematosus) and sometimes on the dorsa of the hands and forearms. However, the hallmark of the syndrome is the consistent proportionate pre- and postnatal growth retardation accompanied by dolichocephaly, and predisposition to a wide variety of malignancies. Recurrent infections (otitis media and pneumonia), chronic pulmonary disease, diabetes mellitus and learning disabilities are common.

• **Werner syndrome **(AR) has a few clinical features of premature aging in common with RTS. Werner syndrome is also known as segmental progeria of the adult, as the cardinal signs (*i.e*. bilateral cataracts, short stature, premature greying, a "bird-like" facies and skin changes) appear after the age of ten. Skin changes include scleroderma-like lesions on acral areas, with mottled hyperpigmentation, telangiectasias, subcutaneous calcification and ulceration. Increased risk of mesenchymal cancer, OS included, has been recorded.

• **Fanconi anemia **(AR) presents with skin pigmentary changes occurring in 55% of the patients, characterised by generalised, dusky, olive-brown pigmentation that is most intense on the lower part of the trunk, in the flexures, and on the neck (85% of patients) [17]. Short stature (51%) and upper limb malformations (43%) are signs in common with RTS. Pancytopaenia typically presents in the first decade. Malformations of the eyes, kidneys and urinary tract, ear, heart, gastrointestinal system, oral cavity, and central nervous system may be present. The patients may also suffer from hearing loss, hypogonadism and developmental delay.

• **Ataxia-telangiectasia **(AR) patients show a combination of progressive cerebellar ataxia, severe combined immunodeficiency (affecting mainly the humoral immune response) and oculocutaneous telangiectasia. Onset, related to ataxia, usually occurs first when the child begins to walk. Cutaneomucosal telangiectasias, which become apparent between the ages of 3 and 7 years, are first seen on the face. These then extend to the neck, the dorsa of the hands and feet, and to the antecubital and popliteal areas. Growth delay is also relatively frequent. Cancer predisposition, particularly to leukaemia and Hodgkin's lymphoma, has been noted.

• **Cockayne **syndrome patients develop a photodistributed erythema, atrophy, and hyperpigmentation. They may simulate RTS, but later develop typical facies, limb abnormalities, wasting and neurological manifestations between the ages of 1 and 2 years. Diagnosis is based on clinical findings: poikiloderma, dwarfism, mental retardation, pigmentary retinopathy, blindness and conduction hearing loss.

## Molecular diagnosis and genetic counselling

Molecular diagnosis is currently the only tool available to a subgroup of RTS patients that can provide them with a basis for adequate genetic counselling, and particularly a recommendation for cancer surveillance for those cases in whom RST II has been confirmed by a molecular test.

The *RECQL4 *scan is a complex procedure due to the need of both DNA and RNA to combine genomic PCR sequencing with RT-PCR methods, which detect splicing and other unique mutations common in RTS. Using RNA analysis, we have identified a homozygous 11 bp deletion in intron 8, which results in intron retention, frameshift and premature termination, and which would not have been detected by standard genomic PCR sequencing [84]. It is worth noting that nearly one third of all the identified *RECQL4 *mutations are splicing mutations (Fig. 3), often not canonical, *i.e*. escaping detection by standard genomic analysis. In all cases in which we were able to analyse the transcripts, valuable information on the type of mutation and predicted effect could be obtained [26,30]. Combined western blots using a C-terminal and a N-terminal antibody are warranted in order to detect whether or not truncated proteins are produced and to translate this information into clinical management of the patients.

RTS is an autosomal recessive genetic disease. Therefore, patients with a consistent diagnosis of RTS, along with their parents and siblings, should be referred for genetic counselling to ensure early identification and treatment of syndrome-associated manifestations. Special attention is needed for cancer surveillance. Construction of the family tree allows the identification of parental consanguinity (third cousins or closer) and of family members who may be affected or at risk of being carriers of a *RECQL4 *mutation.

As with all recessive conditions, both parents of the proband are obligate heterozygotes for a disease-causing mutation. The siblings have a 25% chance of being affected, a 50% chance of being asymptomatic carriers and a 25% chance of being non-carriers.

Heterozygous carriers of a *RECQL4 *mutation are asymptomatic.

## Cytogenetic studies and chromosomal instability

It has been noted that chromosomal instability of RecQ helicase mutant cells is a secondary manifestation of primary defects in replication and repair functions [55]. Difficulties in establishing whether this cellular phenotype might be a useful adjunct to clinical diagnosis are due to: i) the description of chromosomal instability in RTS patients before the *RECQL4 *molecular test became available, making uncertain in these cases the relationship of the observed phenomenon with RTS caused by the RECQL4 defect; ii) the variability in the applied procedure as either spontaneous or induced chromosomal instability was monitored and iii) the variability in the cells used for testing, which include lymphocytes, fibroblasts and lymphoblastoid cell lines. The lack of a standardised method accounts for the conflicting literature data and precludes drawing any standardised guideline for diagnostic purposes. Yet, a few points can be noted. Independent of the method and target cell, chromosomal instability, whenever found, appears to be distinctive and is mainly represented by mosaic aneuploidies and isochromosomes [80]. Multiple evidence for *in vivo *mosaicism of trisomy 8 and/or 7 and/or 2 [13,19,30,46,98,99] has been found, even in the absence of increased spontaneous chromosomal instability [27]. Among structural chromosomal abnormalities, isochromosomes (mainly of the same chromosomes that were found to be trisomic, namely 8, 7 and 2) have been frequently observed and appear to be quite characteristic for this syndrome [13,19,30,99,100]. Mouse embryonic fibroblasts have confirmed this chromosomal instability pattern displaying an overall aneuploid phenotype and a significant increase in premature centromere separation [68]. This latter sign shows a failure of correctly segregating sister chromatids [61]. This may reflect the action of RECQL4 in the N-end rule pathway [57]. While the frequency of breaks was generally normal on lymphocytes from tested RTS cases [13,27,46,98], with a few exceptions [30,101], multiple spontaneous breaks have been observed on skin fibroblasts [6,35,46]. *In vivo*, chromosomal instability may drive neoplastic transformation of cancer stem/progenitor cells that are especially sensitive to the effect of *RECQL4 *mutations. This may be true for cells of the mesenchymal compartment that give rise to the tumours most frequently developed by RTS patients. At the level of the organism, chromosomal instability is an epiphenomenon, primed by the multiple defective RECQL4 functions and possibly modulated by the nature and combination of the two *RECQL4 *mutations. At the somatic level, however, it is (together with the *RECQL4 *point mutation) a powerful co-instigator of tumourigenesis.

As compared to chromosomal instability features of other RECQ helicase defects, RTS has apparently no overlap with Bloom's syndrome. No increase in sister chromatid exchanges (SCEs), which is the characteristic sign of Bloom cells, has been recorded in RTS cells [14,27,89]. RTS resembles Werner syndrome with respect to a few clinical features and the increased predisposition to mesenchymal tumours. However, the spectrum of chromosomal rearrangements appears more restricted in RTS than in Werner cells where the "variegated translocation mosaicism" is enhanced by multiple pathways, including telomere erosion and chromosome end fusions [56]. On the other hand, numerical CIN is typical of RTS and, as in the mosaic variegated aneuploidy syndrome [102], indicates a failure in the mitotic spindle checkpoint.

## Sensitivity of RTS cells to genotoxic agents

Normal results of nucleotide excision repair study, as measured by the functional unscheduled DNA synthesis assay, were found on RTS lymphocytes with an unknown [27] or known *RECQL4 *mutational status [35].

Conflicting results have been obtained on the sensitivity of RTS cells to UV irradiation [6]. These showed either decreased DNA repair [103,104] or a normal response [4,16,27,105]. Inconsistencies were also found among studies of the response to ionizing radiation (IR) [4,106], possibly because of the use of different experimental systems, fibroblasts or lymphocytes, and due to lack of information on the *RECQL4 *status. This may explain part of the heterogeneity of the syndrome. A more recent study on Recql4 KO mouse embryonic fibroblasts using the colony survival assay demonstrated normal sensitivity to both UV and IR [67]. The same result was obtained using a cytotoxicity survival test on human RECQL4-deficient fibroblasts [16]. Cultured lymphocytes from a patient and her mother exposed to mitomycin C and diepoxybutane did not show increased sensitivity to the dialkylating agents [27]. However, a defect in S phase arrest was found in human RECQL4-deficient fibroblasts using FACS (Fluorescent Activated Cell Sorter) analysis [107]. Only recently, a systematic study on the sensitivity to genotoxic agents of primary fibroblasts from 10 RTS patients carrying two deleterious *RECQL4 *mutations has been carried out using the colony survival assay [108]. Results show increased sensitivity to agents that interfere with DNA replication, such as hydroxyurea, camptothecin and the anticancer agent doxorubicin, in line with the role of RECQL4 helicase in DNA replication [55,64]. The same study demonstrated modest sensitivity to agents such as UV and IR and cisplatin that predominantly cause damage that requires DSB repair or nucleotide excision repair mechanisms (NER). Another study showed no sensitivity of primary RECQL4-deficient fibroblasts to any of the above mentioned genotoxic agents nor to agents such as hydrogen peroxide which induces oxidative DNA damage via reactive oxygen species [16], in contrast to previous findings [74]. How can one reconcile these results with the implication of the multifunctional RECQL4 protein in the repair of exogenous damage by homologous recombination [69,70], NER [75] and response to oxidative stress [73,74]? The knowledge of the molecular mechanisms underlying the differences in response of RECQL4-deficient cells to different genotoxic agents will provide clear-cut answers.

## Management and treatment

Patients should be managed by a multidisciplinary team, which includes a dermatologist, an ophthalmologist, an orthopaedic surgeon and an oncologist. All RTS patients should be offered long term follow-up.

Treatment includes the use of pulsed dye laser photocoagulation to improve the telangiectatic component of the rash [109], surgical removal of the cataracts and standard treatment for affected individuals who develop cancer. Surveillance includes annual physical examination by clinicians aware of the natural history and the need for precise long-term follow-up, including careful osteoarticular monitoring for detection of a bone tumour, skin monitoring for lesions with unusual colour or texture and eye examination.

RTS can be considered among the systemic diseases associated with early-onset periodontitis. In the absence of other common secondary features of the syndrome, dental radiographic screening is recommended in the diagnostic work-up of suspected patients. Short root anomaly (rhizomicry) has been associated with an increased tendency to root resorption: thus all factors capable of enhancing further loss of the apical structure of the affected teeth, ranging from masticatory stress, fixed prostheses, caries or periodontal disease, should be avoided [18].

In RTS patients with respiratory symptoms, pulmonary function tests and computed tomography should be considered in the further work-up for bronchitis and bronchiectasis [35].

At diagnosis a skeletal survey of the long bones at infancy has been recommended since underlying skeletal dysplasia may impair subsequent diagnosis of osteogenic sarcoma [19]. It is also recommended to perform complete dermatologic screening for cutaneous tumours and to advise patients to use sunscreens.

A debatable issue is the potential risk of radiation exposure from radiologic screening for osteosarcoma in *RECQL4 *mutation-positive RTS patients. Data on modest sensitivity of RECQL4-deficient fibroblasts to DNA damaging agents (including UV and ionizing radiation [108] do not argue for or against screening. A study on the response to therapy of osteosarcoma in patients with RTS indicated that these patients, even if affected by a chromosomal instability disorder, just as patients with Ataxia-telangiectasia, Fanconi anaemia and xeroderma pigmentosum, do not present the same level of sensitivity to genotoxic agents. For this reason they should be treated initially with conventional doses. However, caution and careful clinical observation is warranted for monitoring for enhanced doxorubicin sensitivity and side effects in the form of mucositis in RTS patients [50]. By contrast, cisplatin (to which RECQL4-deficient fibroblasts are less sensitive [108] can replace doxorubicin as an active chemotherapy agent as it has been shown to cause no apparent increased toxicity [50].

Successful umbilical cord blood stem cell transplantation has been performed in one patient with RTS and combined immunodeficiency [85], while an allogenic bone marrow transplantation has been carried out in another case of RTS with myelodysplastic syndrome [36].

## Prognosis

Although some clinical signs suggest precocious aging, the patients' lifespan is not altered, provided that the neoplastic disease is diagnosed and treated in time.

The histological response of the OS lesions to standard chemotherapy and the clinical outcome in the presence of OS are similar in RTS and non-RTS patients, with a five-year survival rate of 60-70% [50].

## Conclusions and perspectives

One main question that remains open is whether the clinical presentation of RTS as diagnosed by the prototypic sign, *i.e*. poikiloderma, might raise in itself a dichotomy between juvenile cataract and radial ray defects, which would lead to splitting of the syndrome in different subsets with a distinct genetic control. So far, the *RECQL4 *locus has been clearly associated with skeletal defects and susceptibility to cancer, mainly osteosarcoma and skin cancer, while the occurrence of bilateral cataract is not an obligatory sign of *RECQL4*-positive patients. Identification of a second locus accounting for up to one third of clinically diagnosed RTS cases should confirm the locus heterogeneity of the syndrome directing the laboratory flow-chart towards screening of the major *RECQL4 *mutations or the, as yet unknown, minor gene(s). However, even if this goal is reached it is unlikely that the entire clinical expressivity of RTS would be solved. Among clinical sub-entities within the wide spectrum of RTS, Clericuzio type poikiloderma with neutropaenia has been shown to lack *RECQL4 *mutations and has been just recognized to be genetically distinct from RTS [94-96]. The other less defined presentations, broadly defined as variant or atypical [22,97], might result from rare combinations of *RECQL4 *mutations, which have so far escaped detection due to technical reasons or due to involvement of novel genomic or epigenetic mechanisms.

Also, RTS patients who are defined by a positive *RECQL4 *test often have been characterised only for one *RECQL4 *mutation, with a second mutation being undetectable by the standard mutation scan. A challenging issue is the genotype-phenotype correlation in RTS patients: genomic identification of both mutations is insufficient to predict the consequences of the mutations and should be corroborated by transcript analysis and the use of antibodies directed towards the N- and C-terminal parts of the protein. Future studies elucidating the differences in response of RECQL4-deficient cells to different genotoxic agents may provide insights into the comprehension of the molecular basis of RTS. A full comprehension of the defective cellular pathways is of upmost relevance to assess the cancer risk of the affected individuals and to offer them careful surveillance. The recent follow-up of RAPADILINO patients who developed either osteosarcoma or lymphoma [81] has changed the view on predisposition to cancer and cancer types underlined by RECQL4-associated diseases. Quite striking in this regard is the just reported finding of the first out of 24 described patients with BGS who developed a NK/T-cell lymphoma [82]. Sharing of both mutations by patients with distinct syndromes of the RECQL4 spectrum may be more frequent than has been shown thus far and such situations may pave the way to the discovery of modifier genes. Other open questions are the putative cancer risk of unaffected carriers of a *RECQL4 *mutant allele and the involvement of somatic *RECQL4 *mutations in sporadic osteosarcoma, in spite of the fact that this possibility has been excluded by the only study which has so far addressed the issue [110].

## Abbreviations

(RTS): Rothmund-Thomson syndrome; (OS): Osteosarcoma; (MFH): Malignant fibrous histiocytoma; (SCC): Squamous cell carcinoma; (OSMC): Osteosarcoma multicentric; (DSB): Double-strand breaks; (BER): Base excision repair; (ROS): Reactive oxygen species; (UV): Ultraviolet; (XPA): Xeroderma Pigmentosum Group A; (CIN): Chromosomal instability; (RAPADILINO): Radial hypoplasia, Patella hypoplasia and cleft or Arched palate, DIarrhoea and dislocated joints, Little size and limb malformation, slender Nose and nOrmal intelligence syndrome; (BGS): Baller-Gerold syndrome; (SNPs): Single Nucleotide Polymorphisms; (EB): Epidermolysis bullosa syndrome; (PN): Poikiloderma with Neutropaenia; (KS): Kindler syndrome; (SCEs): Sister chromatid exchanges; (FACS): Fluorescent Activated Cell Sorter; (NER): Nucleotide excision repair.

## Consent

The authors are grateful to the families for their cooperation. Written consent for publication of the clinical pictures was obtained from the patients' parents.

## Competing interests

The authors declare that they have no competing interests.

## Authors' contributions

LL designed the structure of the review that has been drafted together with LV and GR. GR took care of the clinical description, differential diagnosis and cancer, LV reviewed the mutations and their distribution. All authors read and approved the final manuscript.

## Supplementary Material

Additional file 1 **Main *RECQL4 *recurrent mutations**. The overview provides for the three recurrent mutations: c.1573delT (red), c.1390+2delT (blue) and c.2269 C>T (green) the associated second mutation, the ethnic background of the carrier patients and the literature references. In case of c.1573delT an associated SNP is also recorded. Click here for file
