# Supplementary material for: Rothmund-Thomson syndrome
Source: Orphanet J Rare Dis. 2010 Jan 29;5:2. doi: 10.1186/1750-1172-5-2 (PMC2826297; doi:10.1186/1750-1172-5-2)
Supplement: Additional file 1 — Main RECQL4 recurrent mutations. The overview provides for the three recurrent mutations: c.1573delT (red), c.1390+2delT (blue) and c.2269 C>T (green) the associated second mutation, the ethnic background of the carrier patients and the literature references. In case of c.1573delT an associated SNP is also recorded. [file 1750-1172-5-2-S1.PDF]

Table 1: Recurrent mutations of RECQL4 gene and associated haplotypes. RTS (Rothmund Thomson syndrome), RAPA (RAPADILINO), BGS (Baller Gerold syndrome)

Syndrome	First Mutation	SNP	Second Mutation	Country	Refs
RTS	c.1573delT	c.1568 G>C	c.1391-1 G>A	England Ireland	89
RTS	c.1573delT		c.1391-1 G>A	Mexico- USA	13,5
RTS	c.1573delT		c.2269 C>T	USA (Arizona)	9,5
RTS	c.1573delT		c.2269 C>T	n.d.	5
RTS	c.1573delT	c.1568G>C	c.2059-1G>T	France	30
RTS	c.1573delT		c.3270delG	n.d.	5
RTS	c.1573delT		c.3523C>T	n.d.	5
RTS	c.1573delT	c.1568 G>C	c.3061C>T	Hungary	90
RTS	c.1573delT		c.84+6del16	England	87,88
RTS	c.1573delT		c.2461C>T	n.d.	87,88
BGS	c.1573delT		c.3061C>T	Belgium	77,79
RAPA	c.1573delT		c.2091T>C	Finland	76

Syndrome	First Mutation	Second Mutation	Country	Refs
RAPA	c.1390+2delT	c.806G>A	Finland	76
RAPA	c.1390+2delT	c.1390+2delT	Finland	76
RAPA	c.1390+2delT	c.1390+2delT	Finland	76
RAPA	c.1390+2delT	c.1390+2delT	Finland	76
RAPA	c.1390+2delT	c.1390+2delT	Finland	76
RAPA	c.1390+2delT	c.1390+2delT	Finland	76
RAPA	c.1390+2delT	c.1390+2delT	Finland	76
RAPA	c.1390+2delT	c.1390+2delT	Finland	76
RAPA	c.1390+2delT	c.1390+2delT	Finland	76
RAPA	c.1390+2delT	c.3214A>T	Finland	76
RAPA	c.1390+2delT	c.3271C>T	Finland	76
RAPA	c.1390+2delT	c.3599_3600delCG	Finland	91

Syndrome	First Mutation	Second Mutation	Country/ethnicity	Refs
RTS	c.2269 C>T	c.2269 C>T	USA (Arizona)	9,5
RTS	c.2269 C>T	c.1573delT	USA (Arizona)	9,5
RTS	c.2269 C>T	c.1573delT	n.d.	5
RTS	c.2269 C>T	c.3072-3073delAG	n.d.	5
RTS	c.2269 C>T	c.1568delG	Caucasian	16
RTS	c.2269 C>T	c.1048_1049delAG	n.d.	87,88
RAPA	c.2269 C>T	c.1885del4	Finland	76
RTS	c.2269 C>T	c.1048_1049delAG	n.d.	5
